# Positive effect on spinal fusion by the combination of platelet-rich plasma and collagen-mineral scaffold using lumbar posterolateral fusion model in rats

**DOI:** 10.1186/s13018-019-1076-2

**Published:** 2019-02-06

**Authors:** Jen-Chung Liao

**Affiliations:** Bone and Joint Research Center, Department of Orthopedic Surgery, Chang Gung Memorial Hospital, Chang Gung University, No. 5, Fu-Shin Street, Kweishian, Taoyuan, 333 Taiwan

**Keywords:** Platelet-rich plasma, Platelet-poor plasma, Lumbar posterolateral fusion, Rat

## Abstract

**Background:**

Platelet-rich plasma (PRP) is autologous in origin and contains a high concentration of platelets which is a source of various growth factors. Previous studies have suggested that PRP has a positive effect in accelerating fusion by an autologous bone graft in a lumbar fusion. The role of PRP on artificial bone grafts in spinal fusion remains controversial. In this study, positive effect on spinal fusion by PRP was hypothesized; in vitro and in vivo studies were designed to test this hypothesis.

**Methods:**

PRP was produced from peripheral blood of Sprague-Dawley (SD) rats. A lumbar posterolateral arthrodesis model was used to test the efficacy of PRP on spinal fusion. Thirty SD rats were divided into three groups by different implants: the PRP group, PRP plus collagen-mineral carrier; the platelet-poor plasma (PPP) group, PPP plus collagen-mineral carrier; and the control group, collagen-mineral only. Spinal fusion was examined using plain radiographs, micro-computed tomography (micro-CT), manual palpation, and histological analysis. The fusion rate by micro-CT and that by manual palpation in groups were compared.

**Results:**

In the micro-CT results, 16 fused segments were observed in the PRP group (80%, 16/20), 2 in the PPP group (10%, 2/20), and 2 in the control group (10%, 2/20). The fusion rate, determined by manual palpation, was 60% (6/10) in the PRP group, 0% (0/10) in the PPP group, and 0% (0/10) in the control group. Histology showed that the PRP group had more new bone and matured marrow formation.

**Conclusions:**

The results of this study demonstrated that PRP on an artificial bone carrier had positive effects on lumbar spinal fusion in rats. In the future, this composite could be potentially used as a bone graft in humans.

## Background

Achieving a successful spinal fusion remains a fundamental procedure for an unstable spine. For this purpose, an autogenous bone graft is still the gold standard of bone graft, but autogenous bone grafts are limited by the amount of bone available and significant donor site morbidity [1, 2]. An allograft is another alternative; however, it has a limited osteo-inductive property with a higher pseudarthrosis rate and has the risk of disease transmission [3]. To overcome these problems, tissue engineering for bone regeneration including various scaffolds, growth factors, stem cells, or gene-modified stem cells has been proposed as an alternative treatment to replace the autogenous bone graft [4–7].

Bone morphogenetic proteins (BMPs), such as BMP-2 and BMP-7, exhibit bone induction potency and are available commercially and approved by the US Food and Drug Administration (FDA) for clinical use in spinal procedures [8]. Local adverse effects such as a hyper-inflammation reaction and unwanted ectopic bone formation have been reported to be associated with doses currently used [9]. Multipotent mesenchymal stem cells (MSCs) or gene-modified mesenchymal stem cells also show efficacy in stimulating bone fusion [10, 11]. But the results were inconsistent. Some studies have shown bone formation is accelerated by combining MSCs with various scaffolds [12, 13]. Conversely, other literatures reported that only a few MSCs were retained at the transplanted site and the grafting ability was low because of cell excretion and death [14]. Furthermore, preparation of stem cells is not easy and the clinical application is limited.

Platelet-rich plasma (PRP) is a concentration of platelets with a small amount of plasma that can be obtained by the centrifugation of peripheral blood. Several kinds of osteo-inductive growth factors, such as transforming growth factor-β1 and platelet-derived growth factor, are known to be included inside PRP [15]. Many studies examine the efficacy of PRP on bone fusion; however, the results were not consistent [16, 17]. Concentrate PRP fibrin gel is suitable for use inside the bone cavity but not in spinal posterolateral fusion because it is washed away easily [18]. Although the collagen sponge can absorb PRP and is maintained for a time at the long bone defect or spinal fusion area, PRP’s osteo-inductive abilities are not as strong as BMP’s; the fusion results are not desirable [19].

In this study, PRP extracted from peripheral blood of rats was developed by our method, and a carrier which is a composite of collagen/β-tricalcium phosphate (β-TCP)/hydroxyapatite (HA) was used for absorbing PRP in the spinal fusion study. Theoretically, this carrier can absorb PRP to produce osteo-inductive ability in fusion, and β-tricalcium phosphate with hydroxyapatite can provide osteoconductive function. The hypothesis that PRP had a positive effect on bone union was proposed. The purposes of this study were to verify the effects of the bone fusion method by using the combination of PRP and collagen-β-TCP-HA composite and to develop a less invasive method for spinal fusion that can become an alternative for autogenous bone grafting.

## Methods

All the experiments were approved by the Institutional Animal Care and Use Committee of our institution (approval number, 2013122606; valid period, 1 August 2014 to 31 July 2016).

### Preparation of PRP and PPP

#### Calculation of platelet counts in blood, PRP, and PPP

The Sprague-Dawley (SD) rats were subjected to general anesthesia with 2% isoflurane. Each rat had 8 ml of blood taken, and the blood was transferred to a centrifuge tube containing 2 ml of acid citrate dextrose solution to prevent clotting. Each centrifuge tube, containing 10 ml whole blood, was centrifuged at 2000 rpm for 10 min. Subsequently, plasma was collected and then further centrifuged at 4000 rpm for 10 min. The supernatant alone is obtained as platelet-poor plasma (PPP). The precipitated platelet at the bottom of the centrifuged tube with supernatant is collected as PRP. The platelet counts in the whole blood, PRP, and PPP were calculated by a hematology analyzer.

#### The concentration of growth factors in PRP and PPP

The concentration of various growth factors including tissue growth factor-β1 (TGF-β1), bone morphogenetic protein-2 (BMP-2), bone morphogenetic protein-7 (BMP-7), and platelet-derived growth factor (PDGF) in PRP and PPP was measured by an enzyme-linked immunosorbent assay (ELISA) method (R&D Systems, Minneapolis, MN).

### Preparation of collagen-mineral composite combined with PRP or PPP

We accumulated 30 ml of PRP mixed with 3.0 ml of thrombin and 30 mg of calcium chloride to form a platelet gel. Prior to the animal experiment, the experimental material collagen-β-TCP-HA composite (FormaGraft, NuVasive Inc., San Diego, CA) was cut into the appropriate size (about 1 × 0.5 cm) before the surgery with the desired group and were added to the required PRP or PPP.

### Lumbar posterolateral fusion model

The rats in the experiment were anesthetized with 1% isoflurane. After the anesthesia, the rats had their back hair shaved off and they were sterilized with iodine. The fascia was exposed from the dorsal midline of the rat skin. Two separate incisions in the lumbar fascia were made 5 mm from the midline and at the L4–L5 transverse process. The transverse processes were decorticated with a high-speed burr. Then, the collagen-β-TCP-HA-PRP (10 rats), collagen-β-TCP-HA-PPP (10 rats), or collagen-β-TCP-HA (10 rats) composites were implanted on the inter-transverse process space of each side. The fascia at the wound was closed with an absorbable 3-0 suture, and the skin was closed with a non-absorbable 3-0 suture. Bacitracin-neomycin ointment was applied on the wound.

### Radiographic assessment

Plain radiographs of all rat spines were evaluated at the 2nd, 4th, 6th, 8th, and 12th week after index surgery under the same radiographic exposure factors (42 kV, 320 mA, 120 cm, 8 mAs).

### Micro-CT analysis

At 12 weeks, all spines underwent high-resolution micro-CT examinations (NanoSPECT/CT, Bioscan, Washington) at the Molecular Imaging Center of Chang Gung Memorial Hospital. The micro-CT data were collected at 65 kVp and 72 μA; it was reconstructed using a cone-beam algorithm supplied with the micro-CT scanner. Visualization and data reconstruction were performed using the software provided by the system. The micro-CT results were used to determine whether the inter-transverse area was becoming fused or not.

### Manual palpation

After complete radiographic evaluation, all rats were sedated and sacrificed. The lumbar and upper sacrum spines were then harvested. The implanted segment was palpated and twisted. A gross union was identified when there was no motion across the surgical segment.

### Histology analysis

After micro-CT, all specimens were histologically assessed. These specimens were fixed in 10% formalin, decalcified using 10% decalcifying solution HCl (Cal-Ex, Fischer Scientific, Fairlawn, NJ), washed with running tap water, and then transferred to 75% ethanol. A sagittal section along the L4 and L5 transverse processes was made for each specimen. The specimens were embedded in paraffin blocks. The tissue blocks were sectioned at 5 μm and stained with hematoxylin and eosin (H&E) staining and Masson’s trichrome staining. Each section is assessed on the base of the new bone formation between the L4 and L5 transverse processes.

### Statistical analyses

The numerical data was compared with a *t* test. The fusion rate between the groups was compared with a post hoc test. A *p* value of less than 0.05 was considered to be statistically significant.

## Results

All SD rats tolerated surgery well; no rats died before harvest.

### Platelet counts in blood, PRP, and platelet-poor plasma

**Fig. 1:**
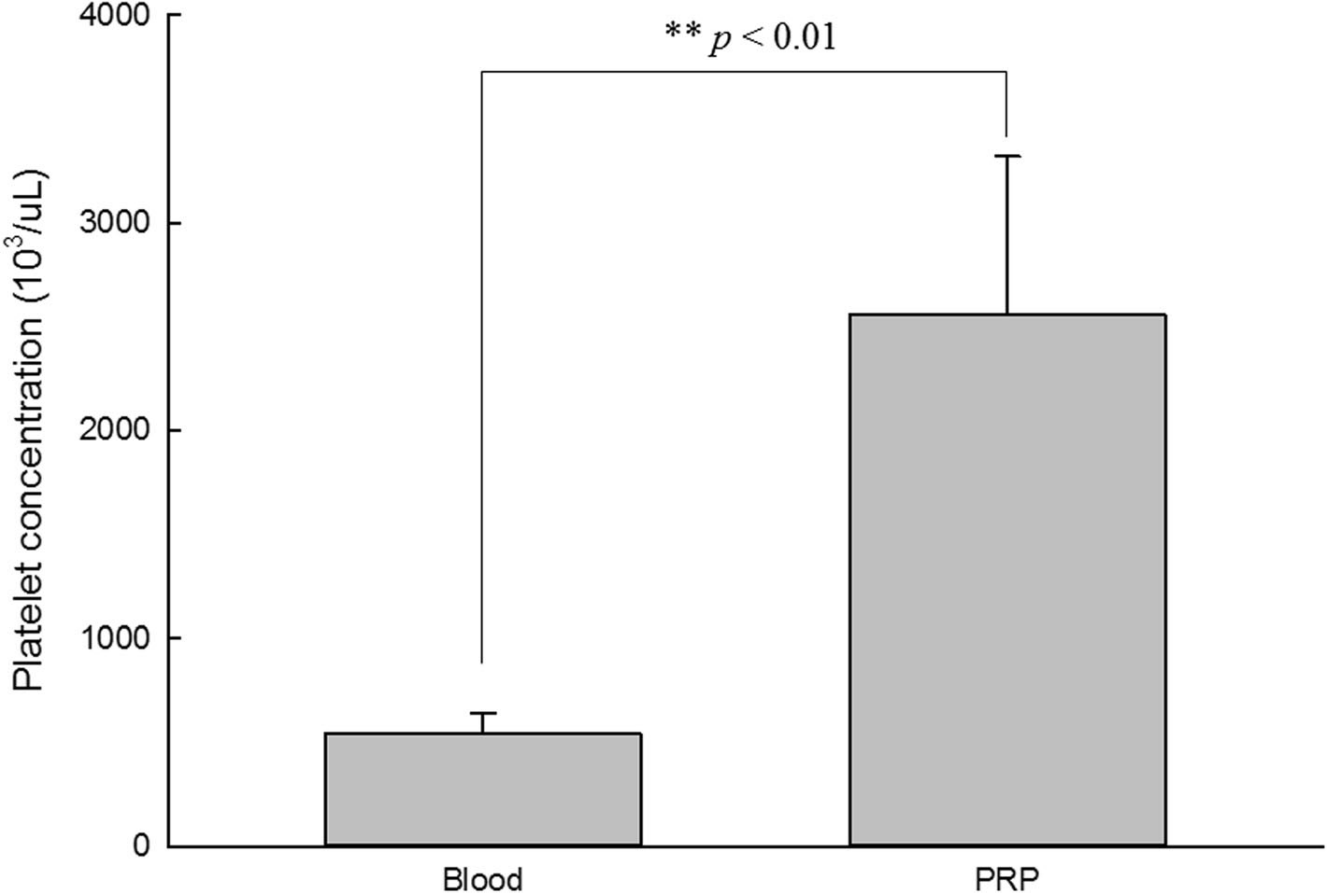
This graph shows the concentration of platelet in PRP is 4.7 times than that in blood. **p value < 0.01

Platelet concentrations in the blood and PRP were measured for each rat. The platelet count in the whole blood was measured as 542.13 ± 99.46 × 10^3^/μl. The platelet count in the PRP was measured as 2557.5 ± 761.56 × 10^3^/μl. The PPP almost could not detect platelet. The platelet count in PRP is 4.7 times higher than that in blood (Fig. 1).

### In vitro study: enzyme-linked immunosorbent assay for growth factors in PRP and PPP

**Fig. 2:**
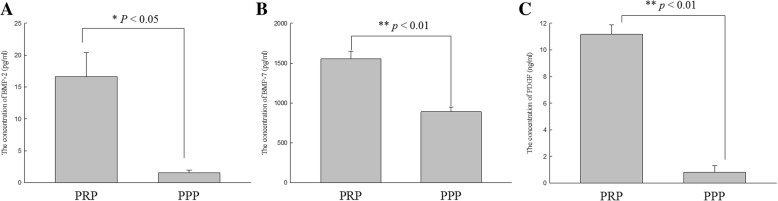
The concentration of the growth factors in PRP and PPP. (**a**) The concentration of BMP-2. (**b**) The concentration for BMP-7. (**c**) The concentration of PDGF. The concentration of BMP-2 and PDGF in PRP was dramatically higher than that in PPP. *p value < 0.05; **p value < 0.01

The concentration of growth factors in PRP and PPP was measured using ELISA. These growth factors include BMP-2, BMP-7, platelet-derived growth factor (PDGF), and transforming growth factor beta 1 (TGF-β1). The concentration of BMP-2 was 16.6 ± 7.6 pg/ml in PRP and 1.6 ± 0.6 pg/ml in PPP. The concentration of BMP-7 was 1555.9 ± 226.9 pg/ml in PRP and 889.1 ± 150 pg/ml in PPP. The concentration of PDGF was 11.2 ± 1.7 ng/ml in PRP and 0.8 ± 1.2 ng/ml in PPP. The concentration of TGF-β1 was below the measurement limits. Figure 2 illustrates the production of these growth factors.

### Radiographic evaluation

**Fig. 3:**
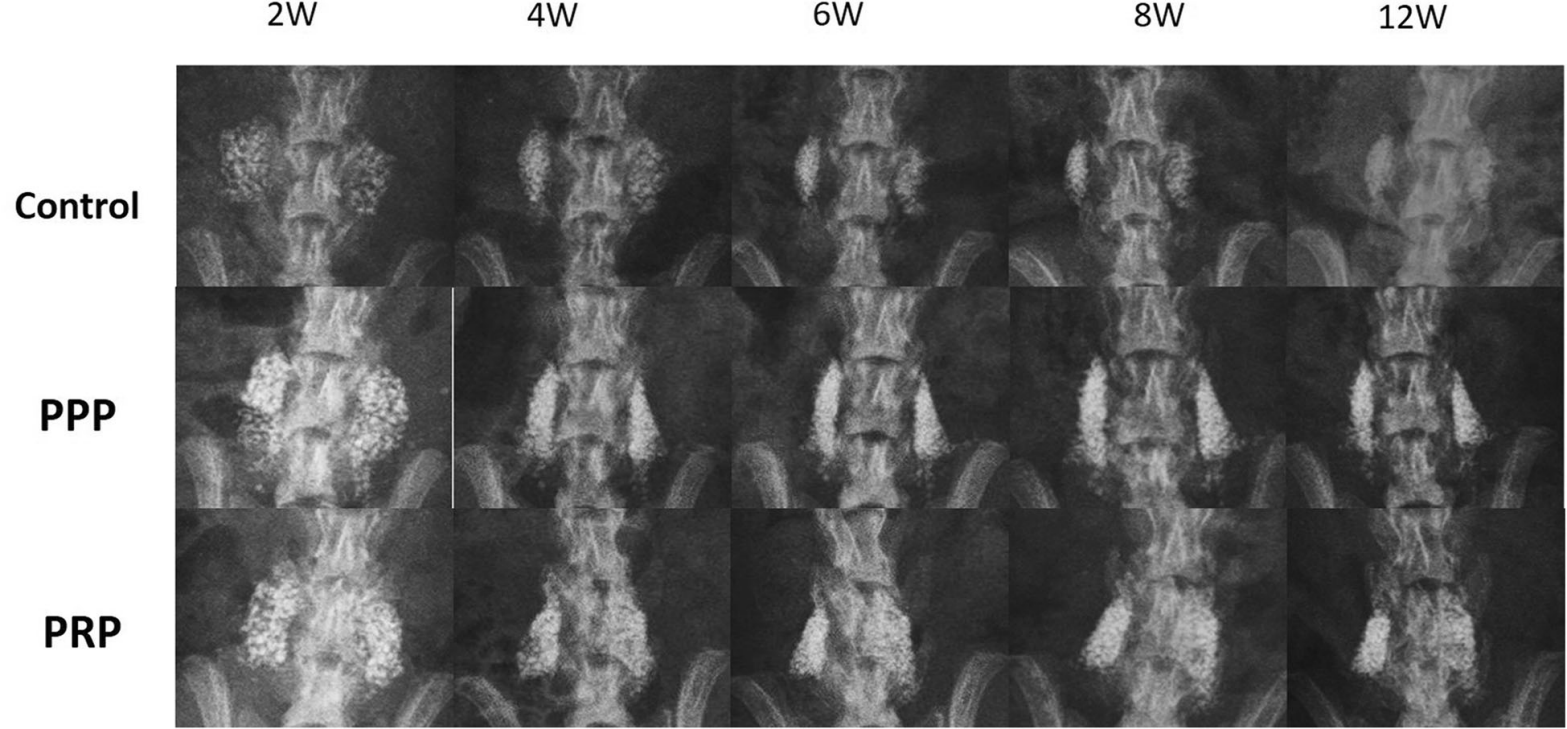
Radiographs of grafted materials in each group at time points of 2 weeks, 4 weeks, 6 weeks, 8 weeks, and 12 weeks. The residual mineral component of the scaffold was still seen in the three groups. But more abundant new bone formation was observed in the PRP group at 12 weeks

Determining a successful fusion from the standard radiographs was difficult because the collagen-β-TCP-HA carrier was not absorbed completely and had a strong radio-opacity by TCP-HA. However, evidence of new bone formation at the margins of the material was present at 12 weeks in the PRP group; the radiographs from the control and the PPP group demonstrated no obvious signs of new bone formation. All carriers of these three groups appeared to undergo shrinkage from the 2nd week to the 4th week, but the shape of these samples seemed not to change from the 4th week to the final follow-up at the 12th week. Typical radiographs at the 2nd, 4th, 6th, 8th, and 12th week following surgery are presented in Fig. 3.

**Fig. 4:**
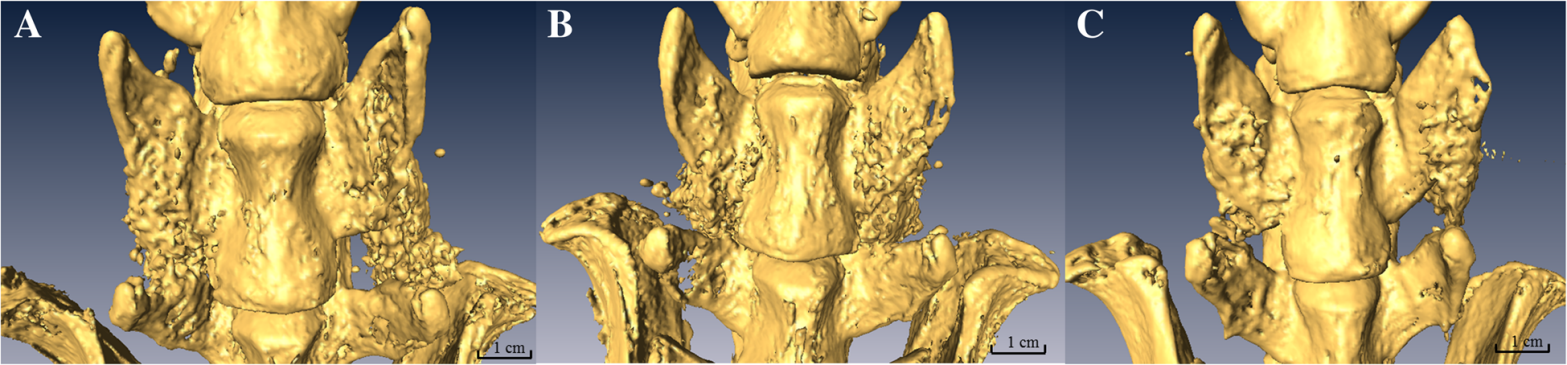
Photos of micro-CT scans from each group. **a** PRP group. **b** PPP group. **c** Control group. The specimen form the PRP group had a stronger fusion mass between the inter-transverse process spaces

By micro-computed tomography (micro-CT) scans, fusion sites with solid calcified materials between the spaces of the transverse process with an uninterrupted bridge were classified as having a radiographic union. The radiographic fusion rates were determined by micro-CT scans; the rates are as follows: the control group 10% (2/20), the PPP group 10% (2/20), and the PRP group 80% (16/20). The PRP group has significantly the greatest fusion rate among the three groups (*p* < 0.001). Figure 4 shows micro-CT photos of these three groups.

### Manual examination

Specimens from the control group showed non-absorbed collagen-β-TCP-HA attached to the inter-transverse process with fibrous tissue. Specimens from the PPP group also revealed some non-absorbed carrier inside the inter-transverse process space, but revealed little bone formation. Specimens from the PRP group demonstrated more bone formation between the transverse process with some residual collagen-mineral composite. Using manual palpation, none in the control group achieved solid fusion (0/10, 0%) as well as in the PPP group (0/10, 0%), but six in the PRP group (6/10, 60%) obtained successful unions. Statistical analysis demonstrated a significantly greater spinal fusion rate in the rats treated with PRP-collagen-mineral composite than in those treated with PPP-collagen-mineral composite or only collagen-β-TCP-HA alone (*p* < 0.001).

### Histological analysis

**Fig. 5:**
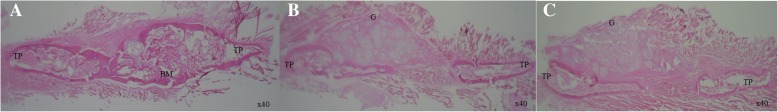
Histological images of 12-week specimens from each group upon hematoxylin and eosin (H&E) staining (original magnification, × 40). **a** PRP group. **b** PPP group. **c** Control group. No inflammatory or lymphatic cells were observed in each specimen of the three groups. The specimen from the PRP group demonstrated more new bone formation. TP transverse process, BM bone marrow, G granules of mineral carrier

**Fig. 6:**
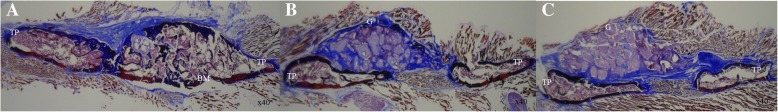
Histological images of 12-week specimens from each group upon Masson’s trichrome staining (original magnification, × 40). **a** PRP group. **b** PPP group. **c** Control group. The specimen from the PRP group revealed more abundant new bone formation with matured fusion mass between the transverse process. The specimens from the PPP group and the control group showed residual unabsorbed scaffolds at the fusion bed. TP transverse process, BM bone marrow, G granules of mineral carrier

No evidence of inflammatory cells or other adverse reactions was observed in any specimen from these three groups in H&E staining (Fig. 5). When using Masson’s trichrome staining, the sections from the control group showed no new bone formation between their transverse processes and only the thick glial fiber material was seen. The PPP group showed similar findings with those of the control group without new bone formation. The sections from the PRP group showed the greatest bone formation and bone marrow formation; it was observed that the sample had successfully bridged the L4–5 transverse process (Fig. 6).

## Discussion

In the present study, the prepared PRP had 4.7 times the count of platelets than in normal whole blood and had two times to ten times the concentration of various growth factors than in PPP. The fusion rates of PRP plus collagen-β-TCP-HA were 60% by manual palpation and 80% by micro-CT examination, which were far superior to the other two groups. By these results, the hypothesis of this study that PRP had a positive effect on bone union could be confirmed. The concentration of platelet in the PRP determines the effect of bony formation. Weibrich et al. demonstrated that the platelet concentration in PRP required for a positive effect on bone regeneration in vivo happens within a limited range; it was between two times and six times higher than the concentration of whole blood [20]. They reported that the lower concentration of platelet in PRP had a limited effect on stimulating bone formation; highly concentrated platelet in PRP had some inhibitory and cytotoxic effects on osteoblast activity. Our prepared PRP had 4.7 times the count of platelets than in normal whole blood, which met the criteria for the bone regeneration described by Weibrich et al. The platelets inside the PRP can release many growth factors. In the current study, PDGF, BMP-2, and BMP-7 concentrations were higher in PRP than in PPP, but the concentration of TGF-β1 was not detected in both PRP and PPP. The higher concentration with PDGF, BMP-2, and BMP-7 in the PRP explained the positive effect on bone formation seen in the PRP group. Early in 1988, Marx et al. tested growth factors in their prepared PRP and showed the appearance of PDGF and TGF-β by the monoclonal antibody staining method [21]. A study from Schmidmaier et al. revealed that PRP could contain various growth factors including BMP-2, BMP-7, PDGF, TGF-β, FGFa, and IGF-1 [22]. However, the amount of these growth factors in PRP seemed inconstant in a different study. In Okamoto’s study, BMP-2 was not detectable in their PRP [14], and TGF-β was not found in PRP by the ELISA method in our study.

The first report about osteogenic ability by PRP preparation in an in vivo bone fusion model was almost 25 years ago; a so-called autologous fibrin adhesion was reported to stimulate early bone consolidation of autogenous cancellous bone during mandibular continuity reconstruction [23]. The first study about PRP in a lumbar spinal fusion model was performed by Li et al. in 2004 [24]. Their experiment showed that when PRP combined with beta tricalcium phosphate granules, it only achieved partial union in a lumbar interbody fusion on a pig. Clinically, adding PRP to autologous bone in a posterior lumbar interbody fusion also did not show any improvement when compared with autologous bone only [25]. Similarly, Cinotti et al. described that PRP was not effective in promoting new bone formation and vascularization in a rabbit lumbar posterolateral lumbar fusion model [26]. In the last 5 years, however, more and more studies have reported positive effects by PRP on spinal fusion. Kamoda et al. performed a study where 40 rats underwent lumbar posterolateral arthrodesis; they found PRP mixed with autogenous bone grafts had a tendency to shorten the period of bone union than those with autogenous bone grafts only [27]. When PRP was prepared in a freeze-dried pattern, which combined with artificial bone, it could achieve an 80% union rate in lumbar posterolateral fusion in a rat model [28]. Clinically, Tarantino et al. reported that 20 patients underwent posterolateral arthrodesis with implantation of a cancellous bone graft soaked with PRP on the right hemi-field and a cancellous bone graft soaked with saline solution on the left hemi-field. They found that the PRP group increased the rate of fusion and bone density using computed tomography scans during the first 6 months after surgery [29]. Similar results were also reported by Imagama et al.. They did a prospective clinical study which consisted of 29 patients who underwent L4/5 posterolateral fusion with PRP/local autologous bone grafts on the right side and local autologous bone grafts only on the left side, and the results revealed that PRP had a positive impact on early fusion in lumbar arthrodesis [30]. Kubota et al. demonstrated the clinical results of 50 patients who underwent instrumented lumbar posterolateral fusion [31]. These patients were separated into two groups: the PRP group (PRP with local bone graft) and the control group (local bone graft only); the results showed that the PRP group had a higher fusion rate, greater fusion mass, and more rapid bone union after surgery.

Fresh PRP was in a liquid condition. An ideal scaffold for PRP binding is the key to achieving a successful spinal fusion or bone union. Okamoto et al. used a scaffold called gelatin β-tricalcium phosphate sponge to absorb PRP in a rat lumbar posterolateral fusion model [14]; the results showed that this PRP sponge was able to achieve a similar fusion rate as the autograft. In the present study, the prepared PRP was soaked on the scaffold that was a composite of collagen, β-TCP, and HA. The collagen is conductive for the deposition of growth factors: β-TCP mimics the trabeculae of cancellous bone and is developed for vascularization and bone ingrowth. HA is usually coated on artificial implants and increases new bone deposition at the interface. PRP was absorbed inside the collagen of the scaffold and slowly released growth factors to achieve osteo-inductive effects on the bone fusion. Other mineral composites provided osteoconductive function. Walsh et al. did a study to demonstrate the effects of collagen/β-TCP/HA scaffolds on rabbits’ spinal posterolateral fusion [32]. Their results showed that new bone formation could be seen around the implanted material; bone mineral density and mechanical testing in the group with the collagen/β-TCP/HA grafts were higher than those in the autograft group. In the current study, we also found new bone formation around the collagen/β-TCP/HA scaffolds on radiographs in the PRP group. From the CT analysis, a thicker bridging mass was also found in the PRP group. In contrast, specimens from the PPP group and the control group also revealed some mineral bridging of the transverse process but most of these bridging masses were interrupted and thinner than those in the PRP group. We believe that the bridging materials of the PPP group and the control group were the remaining inorganic materials of the collagen/β-TCP/HA grafts, not real fused bone, as solid bone masses were not palpable in the harvested specimen in the PPP group and the control group by manual palpation. Furthermore, only specimens from the PRP group could show new bone formation histologically; the other specimens from the PPP group and the control group only showed fibrous tissue without obvious bone formation or calcification because these mineral composites were decalcified during the histologic process.

## Conclusions

In summary, this study demonstrated that PRP contained more abundant platelet counts than whole blood and had various growth factors functioning in vitro. The collagen-β-TCP-HA scaffold adhered with the PRP could successfully achieve spinal posterolateral fusion in the rat model. In the future, PRP combined with a collagen-β-TCP-HA scaffold might provide an alternative to autogenous bone grafts as a fusion material clinically.

## Abbreviations

BMPs: Bone morphogenetic proteins
CT: Computed tomography
ELISA: Enzyme-linked immunosorbent assay
H&E: Hematoxylin and eosin stain
HA: Hydroxyapatite
MSCs: Mesenchymal stem cells
PPP: Platelet-poor plasma
PRP: Platelet-rich plasma
β-TCP: β-Tricalcium phosphate
